# Epidemiology of retinopathy of prematurity (ROP) in Northwest China: a decade-long retrospective single-center analysis (2015–2024)

**DOI:** 10.3389/fped.2026.1874909

**Published:** 2026-07-21

**Authors:** Jing Yang, Le Zhang

**Affiliations:** Department of Ophthalmology, Northwest Women's and Children's Hospital, Xi'an, Shaanxi, China

**Keywords:** epidemiology, incidence rate, treatment rate, retinopathy of prematurity, preterm infants

## Abstract

This study retrospectively analyzes the epidemiology of retinopathy of prematurity (ROP) at a single center to describe incidence trends over the past decade and provide evidence for optimizing ROP screening and treatment. This retrospective database cohort study used the databases from the Northwest Women's and Children's Hospital between 2015 and 2024. The data were stratified into 10 groups according to the screening year (2015–2024) and further categorized into three subgroups based on gestational age (GA): GA <28 weeks, GA ≥28 to <32 weeks, and GA ≥32 weeks. A statistical analysis was performed using SPSS software (version 23.0). A total of 12,640 infants underwent 31,605 screenings (mean 2.44 ± 0.41 per infant). Most screened infants were late preterm (GA ≥32 weeks, 60.42%) or low birth weight (>1,500 to ≤2,500 g, 69.29%). The overall ROP incidence rate was 14.18%, with a treatment-requiring rate of 1.59%. ROP burden decreased markedly with advancing gestational age: for GA <28 weeks, the incidence rate was 72.37% and the treatment-requiring rate was 33.00%; for GA 28–<32 weeks, the rates were 23.04% and 1.39%; for GA ≥32 weeks, the rates were 6.17% and 0.12%. Over the decade, annual screening volume, ROP incidence, and treatment demand showed fluctuating upward trends, suggesting that ROP burden has not declined. Current screening practices appear insufficient. A risk-based strategy is needed: infants with GA <28 weeks require intensive screening, while those with GA ≥32 weeks may safely reduce screening frequency to conserve resources, although ongoing monitoring is warranted given annual fluctuations.

## Introduction

Retinopathy of prematurity (ROP) is a retinal vasoproliferative disease that affects premature infants (1, 2). Globally, around 14.38 million infants are born prematurely every year. The incidence of ROP varies widely across countries, with rates as high as 30% in some regions (3–6). Despite improvements in neonatal care and management guidelines, ROP remains a leading cause of childhood blindness worldwide. The World Health Organization reports that the proportion of severe childhood blindness ranges between 6% and 8% proportion, often associated with conditions like ROP (7, 8).

Reassuringly, blindness can be largely prevented if there is a robust screening program that detects treatment-requiring disease in time. There is still a lack of comprehensive protocols for diagnosing ROP (9). Currently recommended GA limits differ significantly across guidelines and include GA below 30 weeks, below 31 weeks, and below 32 weeks. The guidelines for ROP screening cannot be uniformly implemented, not only due to the complexity of the disease itself but also due to other factors such as variations in national conditions, healthcare resources, clinical environments, and economic capacities across countries. Most included documents define cut-offs for screening as <32 weeks gestational age (GA) or ≤1,500 g birth weight (BW). The highest values were in the Philippines (<35 weeks GA; <2000g) and India (≤34 weeks GA; <2,000 g) (10–13). Over-screening low-risk infants may result in wasted medical resources and increase the psychological and financial burdens on families, while under-screening may lead to missed diagnoses and delayed treatment. For example, a study in Mexico showed that 12% of ROP blind infants had gestational ages (GA) and birth weights (BW) that fell outside the United Kingdom screening guideline criteria (14). Therefore, extensive epidemiological investigations on ROP screening are particularly important.

Nevertheless, epidemiological studies on ROP screening often face substantial obstacles (15–17). For instance, the lack of standardized diagnostic criteria and strong subjectivity in assessments, coupled with the need for physical contact during eye examinations, often leads to parental misunderstanding and refusal of the procedure. This frequently results in loss to follow-up and incomplete data. To address this, we have established a dedicated preterm infant screening clinic with specialized staff responsible for regular follow-ups and recording all examination data for each infant. Our study primarily aimed to summarize and analyze the epidemiological screening characteristics of ROP in Northwest China from a single center over the past decade. This study aims to provide references for the management and policy planning of ROP prevention and treatment.

## Materials and methods

### Study population

All observed subjects met the criteria for the Chinese guideline for ROP, which includes infants with GA < 32weeks (w) and/or BW < 2,000 grams (g) and infants who were suspected to be a risk of ROP (such as those who received long-term oxygen supplementation or infants with serious systemic diseases). The study also included patients who underwent ROP examinations at our hospital within 4–6 weeks after birth or at a corrected gestational age of 31–32 weeks from January 2015 through December 2024. Infants were followed up until retinal vascularization or at a postmenstrual age of 45 weeks, until there was no pre-threshold or threshold disease, with retinal vessels developed into Zone III and the retinopathy regressed. The subjects were stratified into 10 groups according to the screening year (2015–2024 year) and further categorized into three subgroups based on gestational age: GA <28 weeks, GA ≥28 to <32 weeks, and GA ≥32 weeks.

Indications for ROP treatment were based on the current clinical guidelines, including Type 1 ROP (Zone I, any stage with Plus disease; Zone I, Stage 3 without Plus; or Zone II, Stage 2 or 3 with Plus disease) and aggressive posterior ROP (AP-ROP). In this retrospective analysis, all cases of ROP were treated with intravitreal ranibizumab (IVR) 0.25 mg/0.025 mL, administered either once or twice. Only a small number of infants subsequently required laser treatment.

### Data collection

All data, including demographic characteristics, number of infants screened for ROP, number of ROP screening examinations, screening frequency, incidence of ROP (%) (=number of ROP cases/total number of screened infants in the group) × 100%, and treatment-requiring ROP rate (%) (=number of treatment-requiring ROP cases/total number of screened infants in the group) × 100%, were extracted from our electronic medical record database.

For each infant undergoing ROP screening in the Department of Ophthalmology, an ophthalmologist creates and maintains an electronic folder. This folder documents detailed information from each screening session, including patient identification number, name, sex, date of birth, gestational age, birth weight, corrected gestational age, history of oxygen therapy, and ROP stage. If a more detailed medical history was needed, additional information was obtained from the family or by reviewing the patient's comprehensive hospital medical records.

### Ethical considerations

The study procedures strictly followed the ethical principles of the Declaration of Helsinki and the Strengthening the Reporting of Observational Studies in Epidemiology (STROBE) reporting guidelines and were approved by the Research Ethics Board of Northwest Women's and Children's Hospital (No. 2025-070-02). Statistical analysis

Statistical analysis was performed using SPSS (version 23.0). Data were stratified by screening year (2015–2024) and gestational age subgroups (<28 weeks, 28 to <32 weeks, and ≥32 weeks). For each stratum, ROP incidence (%) and treatment-requiring ROP rate (%) were calculated descriptively. No inferential statistical tests were applied, as this study aimed solely to describe decadal trends and distribution patterns of ROP.

## Results

The demographics of all premature infants who underwent ROP screening at our hospital from 2015 to 2024 are presented in Table 1. A total of 12,640 infants (6,962 males and 5,678 females) were included in this retrospective review, accounting for 31,605 screening encounters, with an average screening frequency of 2.44 ± 0.41 per infant. There was no statistically significant difference in gender (*p* = 0.131 > 0.05).

**Table 1:** General characteristics of the patients.

Year	*N*	Male	Female	NS	F
2015	418	247	171	732	1.75
2016	1,098	647	451	2,098	1.91
2017	1,571	997	574	3,226	2.05
2018	1,234	794	440	3,018	2.45
2019	1,572	696	876	3,957	2.52
2020	1,113	636	477	3,114	2.79
2021	1,210	668	542	3,405	2.81
2022	1,178	643	535	3,288	2.79
2023	1,538	762	776	3,851	2.50
2024	1,708	872	836	4,916	2.87
Total	12,640	6,962	5,678	31,605	2.44

N, number of premature infants screened for ROP; NS, number of ROP screening visits; F, frequency of ROP screening.

Information on the study subjects, including their distribution by gestational age and birth weight, are summarized in Table 2. Among the infants screened for ROP, extremely preterm infants (GA < 28weeks) or low birth weight (BW < 1,000 g) infants were least common (accounting for 2.63% and 2.84%, respectively). The largest subgroup was infants with a gestational age ≥32 weeks (7,637 cases or60.42%) or low birth weight (>1,500 to ≤2,500 g) (8,758 cases or69.29%).

**Table 2:** Distribution of study subjects according to gestational age and birth weight.

Year	GA < 28w	GA 28–32w	GA ≥ 32w	BW < 1,000g	BW1,000–1,500g	BW1,500–2,500g
2015	7	155	256	13	123	282
2016	26	482	590	19	313	766
2017	40	718	813	30	312	1,229
2018	30	510	694	28	269	937
2019	45	587	940	48	454	1,070
2020	28	425	660	33	305	775
2021	43	366	801	42	387	781
2022	25	366	787	39	240	899
2023	33	529	976	53	466	1,019
2024	56	532	1,120	54	654	1,000
Total	333	4,670	7,637	359	3,523	8,758

GA, gestational age; BW, birth weight, w, week; g, kilogram; GA 28–32w，28 w ≤ GA＜32 w; BW 1,000–1,500 g 1,000 g ≤ BW < 1,500 g, BW 1,500–2,500 g, 1,500 g ≤ BW < 2,500 g.

From 2015 to 2024, a total of 1,833 infants were diagnosed with ROP, among whom 230 required treatment. The overall ROP incidence was 14.18%, with a treatment-requiring rate of 1.59%. ROP incidence and treatment-requiring rate varied significantly by gestational age, with the highest burden among premature infants GA < 28 weeks (72.37% and 33.00%). In contrast, GA ≥ 32 weeks had markedly lower rates (6.17% and 0.12%). This information is presented in Table 3.

**Table 3:** ROP incidence and treatment-requiring rate stratified by GA (2015–2024).

Year	GA <28 weeks				GA ≥28 to <32 weeks				GA ≥32 weeks			
	ROP (n)	TR-ROP (n)	ROP inc (%)	TR-Rop (%)	ROP (n)	TR-ROP (n)	ROP inc (%)	Tx-R-Rop (%)	ROP (n)	TR-ROP (n)	ROP inc (%)	Tx-R-Rop (%)
2015	6	5	85.71	71.00	26	6	16.77	3.87	13	0	5.08	0.00
2016	16	5	61.54	19.00	100	1	20.75	0.21	47	0	7.97	0.00
2017	28	10	70.00	25.00	115	3	16.02	0.42	38	0	4.67	0.00
2018	22	9	73.33	30.00	132	3	25.88	0.59	37	1	5.33	0.14
2019	33	21	73.33	47.00	124	12	21.12	2.04	66	4	7.02	0.43
2020	17	9	60.71	32.00	106	12	24.94	2.82	42	1	6.36	0.15
2021	29	18	67.44	42.00	105	8	28.69	2.19	47	0	5.87	0.00
2022	19	11	76.00	44.00	82	4	22.40	1.09	49	0	6.23	0.00
2023	29	14	87.88	42.00	100	0	18.90	0.00	61	2	6.25	0.20
2024	46	25	82.14	45.00	186	16	34.96	3.01	71	1	6.34	0.09
Total	245	127	72.37	33.00	1,076	65	23.04	1.39	471	9	6.17	0.12

GA, gestational age; ROP(n), Number of ROP cases; TR, treatment-requiring; TR-ROP (n), number of treatment-requiring ROP cases; ROP inc (%), ROP incidence; TR-ROP(%), treatment-requiring ROP rate.

**Figure 1:**
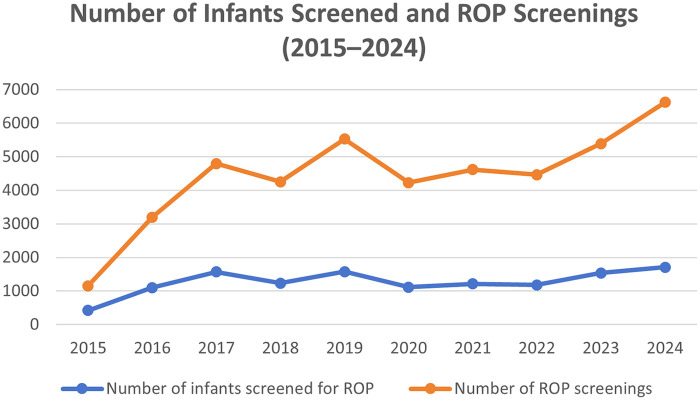
ROP screening volume and screened infants by year, 2015–2024.

 The number screened for ROP significantly increased from 2015 to 2017, with relatively stable trends or mild fluctuations in other years until reaching its peak in 2024 (1,708 premature infants). The fluctuation trend in the number of screening visits closely mirrored the number of infants screened, which remained relatively stable, with minor fluctuations (shown in Figure 1).

**Figure 2:**
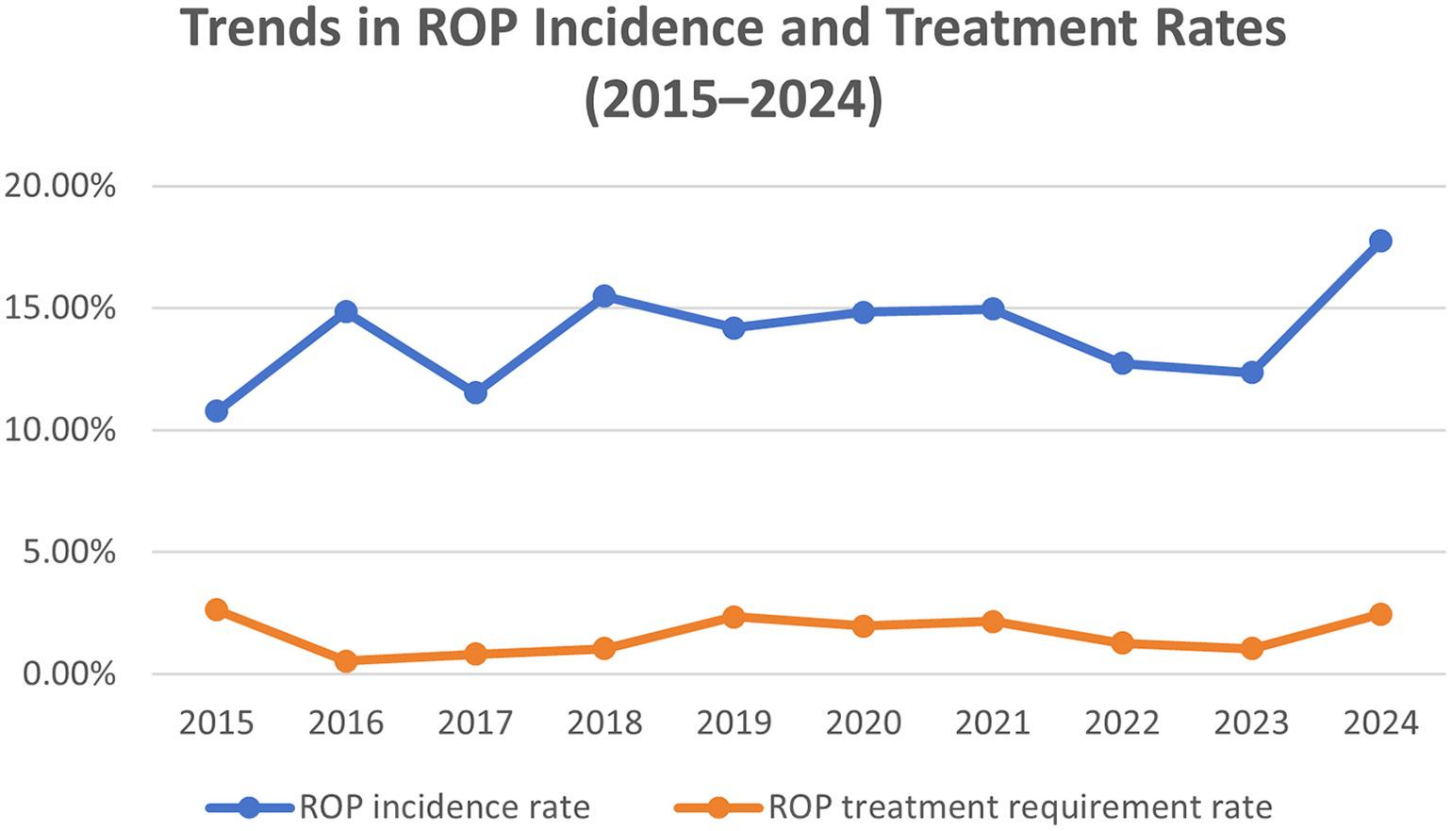
Temporal trends in ROP incidence rate and treatment requirement rate, 2015–2024.

The incidence rate of ROP has shown a fluctuating upward trend, reaching a peak in 2024. The required treatment rate of ROP generally follows the same trend as the incidence rate of ROP, except in 2015 and 2019, when the trends were opposite (shown in Figure 2).

## Discussion

We performed a systematic review on the epidemiology of patients with ROP in a single center. A total of 12,640 premature infants (6,962 males, 5,678 females) were screened for ROP over the past decade. ROP screening volume and the frequency of screening showed an overall fluctuating upward trend. The same trend was also observed in a study by Cindy S (18), who conducted a retrospective review and descriptive analysis of all patients who underwent in-patient ROP screening within a quaternary teaching hospital from 2013 to 2024.

We also found that the proportion of moderate-to-late preterm infants (≥32 weeks) and low-birth-weight infants (1,500 g < BW ≤ 2,500 g) constituted the largest subgroups in the screening cohort, forming the absolute majority of the population undergoing ROP screening. This trend aligns with the gestational age distribution reported in similar cohorts. For instance, Choudhary et al. (4) conducted a prospective hospital-based observational study of 160 neonates over four months and found that the majority of neonates screened were delivered at 33–36 weeks (56.9%), followed by 30–32 weeks (28.8%); 0% were born at <28 weeks. Huseyin Baran Ozdemir et al. (19) also reported similar findings; although the individual risk was higher for very preterm infants, the larger absolute number of late-preterm infants resulted in a greater overall disease burden at the population level. There are multiple potential explanations for these findings, including higher survival rates of extremely low-birth-weight infants due to advances in neonatological care (20–22).

Our results found that the number of extremely preterm infants (GA < 28 weeks) increased from seven cases in 2015 to 56 cases by 2024, with their proportion among screened infants increasing from 1.67% to 3.28% in the same year. This suggests a potential improvement in the survival rate of extremely preterm infants, consistent with the findings reported by Zhao L and Jia C-H (23, 24).

The increase in the volume of ROP screening may also be due to improved screening guidelines that were established around the study period. From 2015 to 2024, the screening guidelines for premature infants in China underwent two revisions. Compared with the 2014 Chinese ROP screening guidelines, the 2021 version expanded the screening criteria by lowering the gestational age threshold for inclusion.

The average screening frequency was 2.44 ± 0.41 per infant, which increased from 1.75 times in 2015 to 2.87 times per infant in 2024. This suggests that, with improvements in economic and healthcare conditions, both pediatricians and the general public may have developed a heightened awareness of ROP.

In our study, overall ROP incidence was 14.18%. These rates were lower than those reported in the meta-analysis by Caberry W. In the study by Yu et al. (25), the overall ROP incidence was 18.8% among 342,005 infants. Similarly, compared with the nationwide multicenter prospective study conducted by Kasia Trzcionkowska et al. (25) in the Netherlands, where ROP incidence was 19.2% in 2009 and 20.4% in 2017, our incidence rate in 2017 (13.18%) remains notably lower. A single-center study by Nasser Shoeibi (19), which screened 9,692 infants over eight years, reported an ROP incidence of 47.8%, which is substantially higher than that observed in our cohort. Additionally, in a retrospective review by Juan Carlos Romo Aguas et al. (26), these discrepancies may largely be attributed to global variations in socioeconomic status, neonatal care quality, and screening inclusion criteria.

From 2015 to 2024, the incidence of ROP showed an overall upward trend. The treatment rate generally followed a similar trend, except in 2015 and 2019 when it diverged from the incidence trend. This is consistent with prior predictions. An examination by Bhatnagar et al. (27), of more than 125,000 patients with ROP from over 23 million births revealed that ROP incidence nearly doubled over the past two decades (28, 29). Similar findings were also reported by Yu CW and Tasman W (29, 30), who noted that, although the growth rate of ROP has plateaued or even decreased slightly in recent years, an overall increase in incidence over the studied period remains apparent.

A strong negative correlation was observed between gestational age and both ROP incidence and treatment-requiring ROP rates in our study. . The incidence rate reached as high as 72.37% and treatment-requiring rate 33.00% in infants GA < 28 weeks, while the rates were only 6.17% and 0.12% in those GA ≥32 weeks. Similarly, in a systematic retrospective meta-analysis encompassing 121,618 infants published between 1985 and 2021, Heladia García et al. (31), reported that the pooled prevalence of any ROP was inversely associated with GA: 61.9% for GA ≤28 weeks, 36.5% for GA ≤32 weeks, and 27.4% for GA >32 weeks. This conclusion is largely consistent with ours, though a notable difference exists regarding preterm infants GA ≥32 weeks.

In our study, the ROP incidence in this group was only 6.17%, whereas Heladia García et al. reported a significantly higher prevalence of 27.4%. This discrepancy may be attributed to differences in study populations. Our study was conducted at a single center and included a homogeneous ethnic group, whereas their research encompassed diverse populations across multiple regions, including North America, Latin America, and the Caribbean, Africa, Europe, Asia, and Oceania, as well as multiple research centers. No significant differences in prevalence were found over the four decades in their study, this trend is also very similar to the pattern we observe in our premature infant screening. Over the ten-year period covered by our study, the incidence of ROP has remained largely stable, with only minor fluctuations.

Larsen et al. (28) reported that infants with GA ≥30 weeks accounted for 33.1%-38.5% of the screened population but only 1.40%-1.42% of treatment-requiring ROP cases. This trend—namely, a very low treatment-requiring rate despite a substantial screening proportion—is consistent with our findings. However, the actual treatment rate in our study was even lower: only 0.12% of the infants with GA ≥32 weeks required intervention. This discrepancy may be attributable to differences in study population, screening criteria, or clinical management protocols between the two settings.

## Conclusions

In conclusion, over the ten-year study period, the annual totals of ROP screenings, ROP incidence, and treatment-requiring ROP cases exhibited fluctuating upward trends. All three outcomes showed a consistent decreasing trend with increasing gestational age. As the survival of preterm infants continues to improve, heightened clinical attention should be directed toward monitoring ROP incidence, particularly among those with GA <28 weeks, who remain at the highest risk. Conversely, for clinically stable infants born at GA ≥32 weeks—in whom the risk is substantially lower—screening intensity could reasonably be reduced. Ongoing re-evaluation and adaptation of ROP screening criteria are essential to minimize unnecessary examinations without compromising diagnostic sensitivity.

## Data Availability

The original contributions presented in the study are included in the article/Supplementary Material; further inquiries can be directed to the corresponding author.
